# The Hunger for Salt: A Tribute to Derek Denton and Jay Schulkin with an Updated Collection of Papers on Salt Appetite

**DOI:** 10.3390/nu15102313

**Published:** 2023-05-15

**Authors:** Michael McKinley, Neil Rowland, Micah Leshem

**Affiliations:** 1 Florey Institute & Department of Physiology, University of Melbourne, Melbourne 3010, Australia; 2 Department of Psychology, University of Florida, Gainesville, FL 32611-2250, USA; 3 School of Psychological Sciences, The University of Haifa, Haifa 3498838, Israel

This collection of outstanding papers is a trove for all concerned with salt intake. It presents the most advanced research and thinking on the fundamentals of salt appetite. However, these contemporary accounts are built on the shoulders of the great pioneers and champions of this scientific field. The title of this front piece, “The Hunger for Salt”, is the title of the great scholarly volume in this field, written by Professor Derek Denton (Figure 1) and published in 1982. It is thus with great sadness that we report the recent death of this giant in the field, and here provide a short tribute to him. The majority of this material has been contributed by one author, (MM), who was a student and colleague of DD for 50 years.

From a scientific perspective, Denton was prolific, publishing more than 300 empirical studies relating mostly to body fluid balance and, in particular, salt appetite. In the latter part of his life, he also published work on the neural basis of consciousness and its evolutionary consequences. Perhaps what is less well-known about Denton is his vision and tireless energy in developing support for the establishment of the Howard Florey Institute in Melbourne and serving as its first scientific director from 1971 to 1990. The present-day Florey Institute in Melbourne can be traced back directly to the care, treatment, and then follow-up scientific analysis of a seriously ill patient in 1947 suffering post-operative electrolyte losses and Derek Denton’s desperate attempts to save this patient. His realization that there were large gaps in the knowledge available at that time to provide the necessary theoretical basis of treatment were the seeds that led him to a lifelong quest to understand the physiological and pathophysiological regulation of body fluids and electrolyte balance. The investigation of this patient was reported in Denton’s first scientific paper, a single-authored letter to Nature, within months of his graduation from the University of Melbourne. Soon after, with Dr Victor Wynn, he pioneered the use of flame photometry in analyzing the blood chemistry of acutely ill patients. Their emergency efforts foreshadowed today’s intensive care units.

After working with Professor Macfarlane Burnet in the late 1940s, Derek Denton joined Professor R.D. Wright in the Department of Physiology, University of Melbourne, to develop an experimental animal model that reflected the body fluid deficits that he had observed in his patients. This became the impetus that led to the building of the Howard Florey Laboratories of Experimental Physiology and subsequently the Howard Florey Institute on the University of Melbourne campus which was completed in 1963; in 1971, Denton became its first Director. He and his co-workers at the Institute used sheep with a transplanted adrenal gland in the neck and a parotid fistula to tap body salt and water, elucidating much new knowledge on the mechanisms controlling the secretion of adrenal steroids, especially the salt-retaining hormone aldosterone and the related peptide hormones renin and angiotensin. He also pioneered investigations of the hormonal and ionic mechanisms acting on the brain that generated thirst and the appetite for salt.

Denton greatly encouraged junior scientists and trusted them with the freedom to test their own ideas. He was also very keen for the junior scientists in the Florey to rub shoulders with “scientific stars”. He would recount to us how he was required to attend morning tea when he was a young scientist at the Physiological Laboratory in Cambridge in the early 1950s. There he was able to discuss both scientific and non-scientific matters with the likes of Andrew Huxley, Alan Hodgkin, and Lord Adrian, all Nobel Laureates. This had a lasting effect on him, and he expected daily attendance of all staff at the Florey tearoom where many an experiment was hatched.

Another aspect of the Cambridge tea-room that impressed Dick (as he was known to friends) was the respect and good humor with which contentious matters were discussed. He passed that message on and emphasized the value of tolerance and a diplomatic approach in dealing with colleagues and other scientists, and, indeed, our team operated in a most amicable and collegiate fashion.

As director, he had the foresight to broaden the Institute’s areas of scientific endeavor and attracted outstanding researchers to the Florey to establish research groups in hypertension, reproductive biology, peptide chemistry, molecular biology, and neuroscience. At this time, Denton also wrote “The Hunger for Salt” [1].

After retiring from the directorship in 1990, he reinvented his scientific direction concentrating on brain function using functional magnetic resonance imaging to gain new insights into what he termed instinctive behavioral drives, such as thirst or the need to breathe, published as recently as 2020. Additionally, he journeyed to West Africa to study the effects of dietary salt on the blood pressure of chimpanzees, our closest relative in the animal kingdom, thereby providing crucial insight into the relationship of sodium intake and blood pressure. Derek Denton was a fundraiser extraordinaire, gaining early major support for the Institute from the NIH in the USA and the local NHMRC, as well as private philanthropic support from many sources. His personal touch and close links contributed greatly to this success.

Derek Denton brought to the Florey a constant stream of outstanding scientists from all over the world, Nobel Laureates, whiz kids, and the odd éminence grise who came for either short or more extended stays, and who provided great encouragement and “contacts” for students and staff. He also recognized unconventional but talented individuals who may not have gained straight A’s in their degree courses but were able to develop into excellent scientists. This was a manifestation of his tolerance for the unusual, his sense of humor, caring nature, and respect for diverse opinions. The ambience that Dick created at the Institute was one of grace and style. His unparalleled network of friends and colleagues in the arts, ballet, politics, academia, science, business, and the media throughout the world ensured this. He was honored by many of the major scientific academies and societies of the world, including election as a Fellow of the Royal Society and membership of the Royal Swedish Academy, French Academie des Sciences, U.S. National Academy of Sciences, and was awarded Australia’s highest civilian honor, Companion of the Order of Australia. He worked closely with the Australian Academy in fighting for the rights of scientists persecuted for their views in other parts of the world.

What characteristics marked this outstanding Australian? Enthusiasm for scientific enquiry, a disciplined and logical approach to solving problems, both scientific and organizational, and the sheer willpower to “have a go” at any endeavor no matter how seemingly difficult. As well as a tolerance for different opinions, and the ability to see the “big picture”, his personality was marked by a tenacity and sheer will to win. He never gave up on any pursuit without a hard fight, regardless of the odds. All of these characteristics typified Derek Denton who died at the age of 98 on 18 November 2022.

I thank Mike McKinley and Neil Rowland for their stirring appreciation of Derek ‘Dick’ Denton and his awe-inspiring documentation and analysis of salt appetite, and his inspiration to all of us studying it.

Sadly, since coauthoring one of these papers, Jay Schulkin (Figure 2), friend and colleague, has died (17 March 2023). Jay was a prolific polymath philosopher–scientist, making a marked contribution to our science through his many pioneering collaborations with our colleagues. Jay’s Book, *Sodium Hunger: The Search for a Salty Taste* [2], graces all our shelves.

Here is the link to his eulogy by Peter Sterling: https://www.madinamerica.com/2023/03/tolstoys-hermit-jay-schulkin/ (accessed on 21 March 2023).

Here is another by Kent Berridge and Harvey Grill: https://www.ssib.org/web/memorial-Schulkin.php (accessed on 29 March 2023).

This special Issue is extraordinarily important. The contributors, all leaders in the field, present a modern synthesis of the science of salt appetite in one platform for the first time in decades, during which there have been substantive advances. Moreover, in their papers, the authors reach well beyond an in-depth update; they include fascinating hypotheses stretching the realms of our understanding.

It is notable that the science has remained somewhat hidden from the prodigious investment into the effects of salt on non-communicable disease and the global effort to reduce salt intake. This is odd insofar as salt appetite is the root cause of both of these. As Baumer-Harrison and colleagues clearly state, “Understanding salt preference can help guide interventions aimed at reducing sodium intake” [3].

In ‘Sodium homeostasis, a balance necessary for life’ Bernal, Zafra, Simon, and Mahía provide an incredibly detailed description of the mechanisms orchestrating sodium homeostasis [4]. Body sodium levels must be maintained within a narrow range for the correct functioning of the organism. Sodium disorders include not only hypernatremia, as in diabetes insipidus, but also hyponatremia, as in cerebral salt wasting syndrome. Body sodium levels therefore require a delicate equilibrium to be maintained between sodium ingestion and excretion. The authors present a travelogue accompanying sodium ion into the mouth, docking on the tongue sodium channel of the apical membrane above the tight junction, through to specific brain pathways, organs, nuclei, and loci subserving its reward properties, to humoral pathways to kidney and vascular and cardiac sources of the endocrine cascades which maintain extracellular fluid volume and ultimately regulate behavior, known as sodium appetite.

In ‘Effects of voluntary sodium consumption during the perinatal period on renal mechanisms, blood pressure, and vasopressin responses after an osmotic challenge in rats’, Porcari, Macagno, Mecawi, Anastasía, Caeiro, and Godino share their extraordinary findings on the prescient and widespread effects of maternal, *voluntary*, high sodium intake during pregnancy and lactation, altering their adult offspring cardiovascular, brain, and kidney mRNAs, receptors, and microstructure [5]. They continued to study the effect of a sodium overload challenge on offspring blood pressure responses and found that males had a more sustained increase. Thus, the perinatal availability of a rich source of sodium induces long-term effects modifying renal, cardiovascular, and neuroendocrine responses regulating salt and fluid, with notable sex differences. Might such comprehensive effects suggest early determinants of salt intake in humans?

Further to the determinants of salt intake, in ‘Sodium intake and disease: another relationship to consider’, Baumer-Harrison, Breza, Sumners, Krause, and de Kloet present an innovative, instructive and very thorough analysis upending the consensual, while yet emphasizing the relationship of salt intake and disease [3]. They illustrate how a reciprocal neural relationship of stress and cardiometabolic disease alters the consumption of sodium, leading to their astonishing conclusion of a resulting vicious cycle exacerbating disease. They outline how the neural circuits regulating sodium intake and blood pressure may contribute to pathology, and in turn are influenced by both stress and cardiometabolic disease. The implications of reverse causality to the relationship of cardiometabolic disease and salt intake are intriguing.

Additionally challenging the consensus, in ‘Homeostatic reinforcement theory accounts for sodium appetitive state- and taste-dependent dopamine responding’, Duriez, Bergero, Cone, Roitman, and Gutkin, in a carefully crafted hypothesis, deconstruct the mechanisms of homeostasis into remarkable hypotheses of adaptive learning to address the little-considered *fundamental* question of why salt is consumed beyond its necessity [6]. The authors use their model to reproduce behavioral data and the underlying brain dopamine signaling that plays a key role in signaling potentially need-fulfilling outcomes. Building on these, they show how reinforcement learning is dynamically tuned by homeostatic needs to regulate both sodium hunger and satiety. This paper suggests that appetitive behavior may be driven by reinforcement learning mechanisms tuned by homeostatic needs, an interesting perspective on allostasis.

In ‘Sex differences in salt appetite: perspectives from animal models and human studies’, Santollo, Daniels, Leshem, and Schulkin, for the first time review in detail the essential sex differences in salt appetite [7]. There is a large body of literature on sodium intake in laboratory rats, but the vast majority of these studies have used male rats. However, the limited work conducted in both male and female rats reveals sex differences in sodium preference, intake, needs and disposition. This review describes the neuroendocrine controls of fluid balance and mechanisms underlying sex differences in salt intake, changes in salt intake during pregnancy and the possible neuroendocrine bases of these differences in behavior. Although we do not know how many of these findings can be generalized to men and women, the limited literature on sex differences in human dietary salt intake, in reproduction and with age, a possible wake-up call for the reduction challenge.

In ‘Neurobehavioral mechanisms of sodium appetite’, Rowland focuses on methodology, presenting ecological, physiological and recent neuroscience work involving the unique motivational state of sodium appetite as a backdrop for laboratory protocols for the study of sodium appetite under controlled conditions [8]. Neil focuses on two extant protocols, a sodium-deficient diet (SDD) for at least one week, and accelerated sodium loss using SDD for 1–2 days coupled with the diuretic furosemide. The modality of consumption is also considered, ranging from free intake of concentrated sodium solution to sodium-rich food or gels, and to operant protocols. Describing the pivotal role of angiotensin and aldosterone in sodium appetite, Neil queries whether intake and appetite, in fact, match physiological state. Finally, he recommends that future studies emulate natural conditions in which overconsumption does not occur, using either SDD only as a stimulus, offering a sodium-rich food for the assessment of appetite, or a simple operant task.

This is an excellent comprehensive yet brief and novel synthesis that is unavailable in other recent reviews, and will serve well both experts in the field, physiologists, behaviorists, and other neuroscientists investigating sodium appetite or interpreting this literature.

All the contributors to this Special Issue have expressed their appreciation for the incisive but constructive reviewers, our colleagues in the field, who invested much careful work to vet scientific accuracy and improve our papers. As Editor (ML), I have written to the reviewers in person for permission to thank them by name on our behalf, without reference to the specific papers they reviewed, so that we and journal readers can appreciate their contribution. Our thanks to our colleagues Carina Andrade, Albertino Bigiani, Joel C. Geerling, Andrea Godino, Michael McKinley, Mitchell Roitman, Christopher Rugg and our additional anonymous reviewers.

In sum, the papers in this Special Issue teach us the details of the body’s regulation of salt intake, how to study it best, that mother’s salt intake determines adult offspring physiology, that salt can condition homeostasis, that disease can increase salt intake to exacerbated illness, and that males and females handle salt very differently.

Finally, readers interested in plumbing the depths of this science will find an exhaustive reference list in this Special Issue that details every aspect of salt appetite.

## Figures and Tables

**Figure 1:**
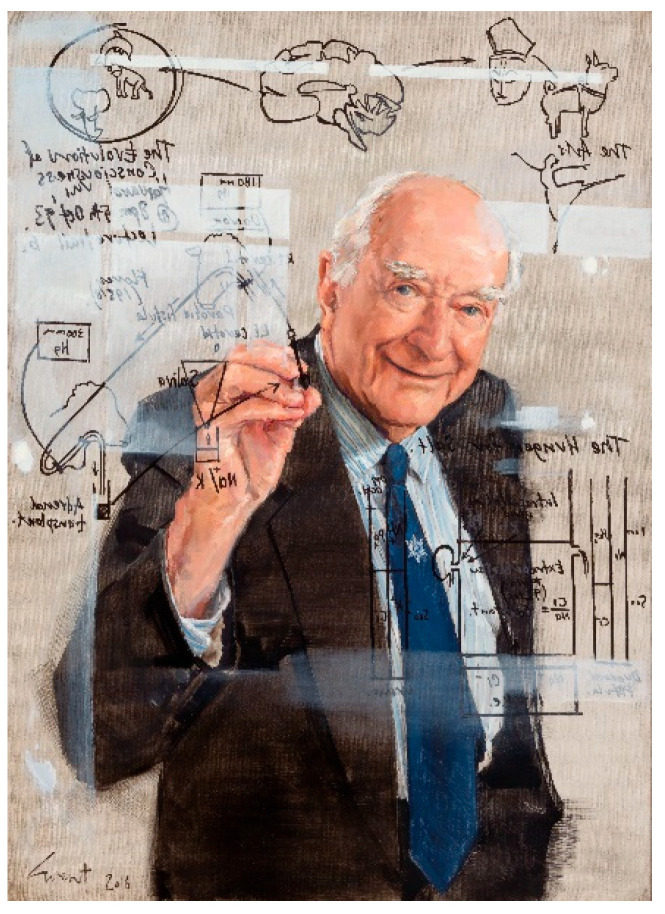
Equation of a life—a portrait of Professor Derek Denton 2016, by Evert Ploeg. National Portrait Gallery of Australia. Commissioned with the assistance of funds provided by Janet Whiting AM, Philip Lukies, and Antonia Syme 2016. © Evert Ploeg.

**Figure 2:**
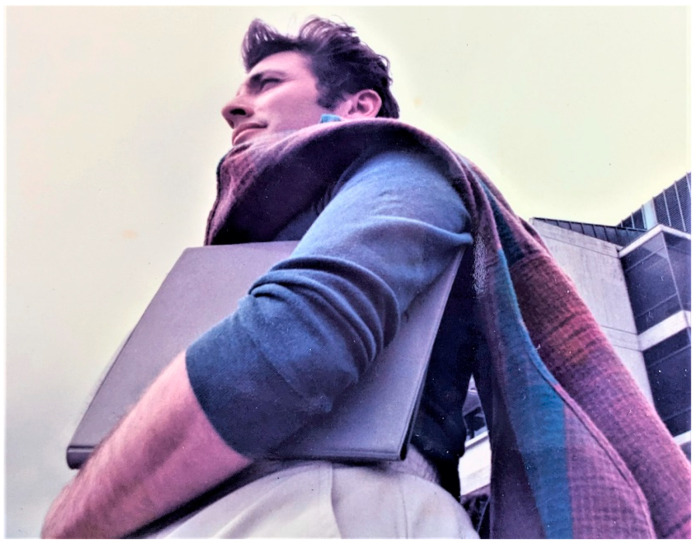
Jay Schulkin, 29.12.1952–17.3.2023.
